# Targeted inhibition of integrin αVβ3 induces cytotoxicity and suppresses migration ability in ovarian cancer cells and tumor spheroids

**DOI:** 10.7150/ijms.103141

**Published:** 2025-02-28

**Authors:** I-Lun Hsin, Ling-Yen Chiu, Jiunn-Liang Ko, Po-Hui Wang, Pei-Ju Wu

**Affiliations:** 1 Institute of Medicine, Chung Shan Medical University, Taichung 40201, Taiwan.; 2 Department of Nursing, National Taichung University of Science and Technology, Taichung, 40640, Taiwan.; 3 Institute and Department of Food Science, Central Taiwan University of Science and Technology, Taichung, 40601, Taiwan.; 4 School of Medicine, Chung Shan Medical University, Taichung 40201, Taiwan.; 5 Division of Medical Oncology, Department of Internal Medicine, Chung Shan Medical University Hospital, Taichung, 40201, Taiwan.; 6 Department of Obstetrics and Gynecology, Chung Shan Medical University Hospital, Taichung, 40201, Taiwan.; 7 Department of Medical Research, Chung Shan Medical University Hospital, Taichung, 40201, Taiwan.

**Keywords:** 3D cell culture, Cell migration, extracellular matrix, Integrin αVβ3, ovarian cancer

## Abstract

Ovarian cancer is a gynecological malignancy that has poor prognosis and high lethality. Integrin αVβ3 is highly expressed in solid cancer cells, including ovarian cancer, and is important in proliferation and cell migration. In this study, we performed two-dimensional (2D) and three‑dimensional (3D) cell culture systems to investigate the potential of integrin αVβ3 as a therapeutic target for ovarian cancer. Inhibition of integrin αVβ3 by *antagonist* cilengitide (CGT) and shRNA significantly reduce the cell viability of ovarian cancer cells. Co-treatment of CGT and cisplatin induced synergistic cytotoxicity in SKOV3 cells. CGT reduced the protein expressions of phospho-FAK, CD44, and PD-L1. CGT reduced mitochondrial membrane potential and induced apoptotic cell death. To mimic the tumor growth in the extracellular matrix, a tumor spheroid formation assay was performed with Matrigel and epidermal growth factor (EGF). CGT reduced the size of spheroids that grew in 50% Matrigel with or without EGF induction. CGT also enhanced the inhibiting effect of T cells on tumor spheroids. The cell migration ability of SKOV3 cells was blunted by CGT by tumor spheroid-based migration assay. This study used 2D and 3D cell models to provide novel insight into ovarian cancer therapy by targeting integrin αVβ3 and suitable cell models for searching integrin αVβ3-targeting drugs.

## 1. Introduction

Ovarian cancer is a gynecological malignancy that has poor prognosis and high lethality 1. Surgery and chemotherapy are the common treatments for ovarian cancer. Bevacizumab and olaparib are the few targeted therapy drugs that approved to treat ovarian cancer 2-4. Therefore, it is important to develop new targeted therapy drugs for ovarian cancer therapy.

Integrins are critical proteins that act as receptor of extracellular matrix (ECM) and induce several signals for cell survival, proliferation and motility 5, 6. Integrin αVβ3 is highly expressed in cancer cells from different solid cancer, including ovarian cancer 7. In recent years, integrin αVβ3 is considered a potential target for cancer therapy 7, 8. Integrin αVβ3 promotes the proliferation of ovarian cancer cells via crosstalk with thyroid hormones and estrogen receptor alpha 9, 10. Anoikis resistance of ovarian cancer cells is triggered by activation of integrin αVβ3 11. Interaction of integrin αVβ3 and vitronectin enhances the cell migration ability of ovarian cancer cells 12, 13. These evidences hint that integrin αVβ3 is a potential therapeutic target to inhibit ovarian cancer progression.

In this study, two-dimensional and three‑dimensional cell culture systems were performed to investigate the potential of integrin αVβ3 as a therapeutic target for ovarian cancer. Inhibition of integrin αVβ3 significantly inhibited cell viability and reduced the migration ability of ovarian cancer cells. This study provided insight into integrin αVβ3-targeting therapy in ovarian cancer and the cell models used to investigate the integrin αVβ3-targeting drugs.

## 2. Materials and methods

### 2.1 Cell culture and chemicals

SKOV3 cells (ATCC, HTB-77) and TOV-21G cells (ATCC, CRL-3577) were obtained from the American Type Culture Collection. SKOV3 cells were cultured in Minimum Essential Medium (MEM) (GIBCO, 41500-034) supplemented with 10% fetal bovine serum and 4.5 g Glucose per L. TOV-21G cells were cultured in 1:1 mixture of MCDB 105 medium (Sigma-Aldrich, M6395) and Medium 199 (GIBCO, 31100035), and supplemented with 15% fetal bovine serum. Cilengitide (22289) was purchased from Cayman Chemical (Ann Arbor, MI). Cisplatin (P4394) was purchased from Sigma (St. Louis, MO, USA).

### 2.2 MTT assay

After treatment with indicated drugs for 48 h, the medium with drugs was removed, and 100 μL of fresh medium containing 0.5 mg/mL 3-(4,5-dimethylthiazol-2-yl)-25-diphenyltetrazolium bromide (MTT; Sigma, M 2128) was added to the wells and incubated for another 3 h. The blue water-insoluble MTT-formazan crystals are solubilized with 100 μl dimethyl sulfoxide (DMSO) (Merck, 1.02931.1000) and the intensity is measured colorimetrically at a wavelength of 570 nm.

### 2.3 Western blot assay

Anti-integrin αV (#4711, Cell Signaling, Danvers, MA, USA), anti-integrin β3 (#13166, Cell Signaling, Danvers, MA, USA), anti-PD-L1 (NBP1-76769, Novus Biologicals, Centennial, CO, USA), anti-phospho-FAK Tyr397 (#8556, Cell Signaling, Danvers, MA, USA), anti-CD44 (#3570, Cell Signaling, Danvers, MA, USA), anti-cleaved PARP (#5625, Cell Signaling, Danvers, MA, USA), anti-cleaved caspase-7 (#9491, Cell Signaling, Danvers, MA, USA), and anti-β-actin (AC-40, Sigma, St. Louis, Missouri) were used to detect the protein expression levels of integrin αV, integrin β3, PD-L1, phospho-FAK Tyr397, CD44, cleaved PARP, cleaved caspase-7, and β-actin. The complete protocol for Western blot assay has been described in a previous publication 14.

### 2.4 Integrin αVβ3 detection by immunocytochemistry and flow cytometry

SKOV3 and TOV-21G cells were seeded onto coverslips in 60 mm plates and then incubated for 16 h. The complete protocol for fixation, permeabilization, and blocking was described in a previous publication 15. The FITC conjugated anti-integrin αVβ3 antibody (MAB1976F, Merck, Darmstadt, Germany) was used to detect the integrin αVβ3 in cells. After overnight hybridization at 4 °C, the cells were DAPI (4',6-diamidino-2-phenylindole) for 30 min at room temperature and observed under a fluorescence microscope.

To investigate the surface integrin αVβ3 expression in SKOV3 and TOV-21G cells, the cells were stained and analyzed by flow cytometry. SKOV3 and TOV-21G cells were seeded onto 60 mm dish and then incubated for 16 h. Both cells were collected by Accutase cell detachment solution (SCR005, Merck, Darmstadt, Germany). Cells were stained by FITC conjugated anti-integrin αVβ3 antibody. The non-fluorescent anti-integrin αVβ3 antibody was used as competing antibody. After staining, the cells were analyzed by flow cytometry.

### 2.5 Apoptosis and mitochondrial membrane potential assay

After treatment of CGT for 48h, the SKOV3 cells were used to investigate apoptosis and mitochondrial membrane potential. Annexin V-FITC Apoptosis Detection Kit (556 547, BD Biosciences, CA, USA) and 5,5,6,6'-tetrachloro-1,1,3,3'-tetraethylbenzimi-dazolylcarbocy-anine iodide dye (JC-1, T3168, Invitrogen, Carlsbad, CA, USA; Thermo Fisher Scientific, Inc., San Jose, CA, USA) were used to analyze the apoptosis and mitochondrial membrane potential. The complete protocols for these analyses are described elsewhere 14, 16.

### 2.6 Tumor spheroid formation and migration assay

SKOV3 cells (1 × 10^3^ cells) were seeded into a well of ultra-low attachment 96-well plate (Corning Inc., Corning, NY, USA) containing 100 μL of culture medium (Minimum Essential Medium supplemented with 10% fetal bovine serum and 4.5 g Glucose per L). After incubation for 96 h, 200 μL of fresh medium containing CGT was added to the well. To examine the effect of ECM on spheroid formation, after seeding for 96h, the Matrigel (BD Biosciences, 354234) was added to the well to lead to spheroid growth in the 50% Matrigel. After the Matrigel form the gel, 100 μL culture medium containing CGT with or without epidermal growth factor (EGF) was added to the well. The spheroids were incubated for another 7 days and observed under inverted light microscopy. The volume of the spheroids was calculated by the formula 0.5 × larger diameter (mm) × small diameter (mm)^2^.

To analyze the effect of CGT on T cells induced cytotoxicity on SKOV3 tumor spheroid, Jurkat T-cells were used to perform the assay. The method of Jurkat T-cells activation by T Cell Stimulation Cocktail (eBioscience) was based on the previous study 17. After 96 h incubation for SKOV3-EGFP tumor spheroid formation (1 × 10^3^ cells/well of ultra-low attachment 96-well plate), the Jurkat T-cells (1 × 10^4^ cells/well of ultra-low attachment 96-well plate) in 200 μL of culture medium in the presence of CGT and 1× T Cell Stimulation Cocktail were added to the well. The fluorescence intensity was analyzed by ImageJ software.

To investigate the cell surface PD-L1 expression, the spheroids with or without treating with CGT for 7 days were removed to eppendorf tube. After washing with PBS, the spheroids were stained by Alexa Fluor 488 conjucated PD-L1 antibody (#25048, Cell Signaling, Danvers, MA, USA) that specific recognizes extracellular domain of PD-L1. After staining, the spheroids were removed to 60 mm dish and observed under inverted fluorescence microscope. The fluorescence intensity was analyzed by ImageJ software.

To investigate the cell migration, the spheroids after treating with CGT for 7 days were removed to the 6-well plate for attachment. The spreading area of SKOV3 cells migrated from the tumor spheroid was measured by ImageJ software at indicated time.

## 3. Results

### 3.1 Integrin αVβ3 inhibition decreases cell viability and increases cisplatin cytotoxicity in ovarian cancer cells

**Figure 1:**
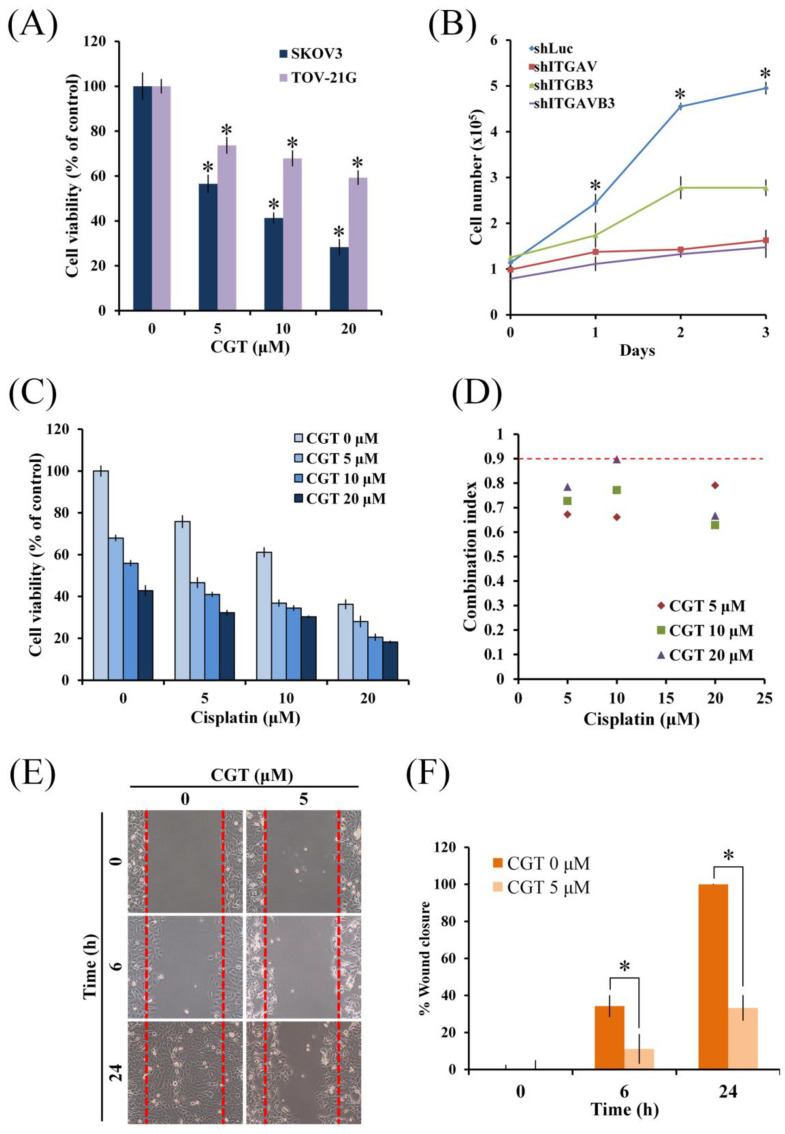
Effect of CGT and knockdown of integrin αVβ3 on cell viability in ovarian cancer cells. (A) SKOV3 and TOV-21G (4 × 10^3^ cells/well of 96-well plate) were treated with various concentrations of CGT (0, 5, 10, and 20 μM) for 48h. Cell viability was analyzed by MTT assay. (B) The indicated cells were harvested by trypsin, stained with trypan blue, and counted under an inverted microscope at indicated times. (C) After combined treatment with CGT and cisplatin for 48h, MTT assay was performed to analyze the cell viability of SKOV3 cells. (D) Combination index of cotreatment of CGT and cisplatin for 48 on SKOV3 cells was calculated by software Compusyn 1.0. The definitions of combination index are synergistic effect (combination index < 0.9), additive effect (0.9 < combination index < 1.1), and antagonism (combination index > 1). (E) Representative images of wound healing of SKOV3 cells with or without CGT treatment. (F) Values of percentage wound closure. The areas lacking cells were analyzed by ImageJ. The symbol '*' indicates P < 0.05.

To investigate the cytotoxic effect of cilengitide (CGT), an antagonist of integrin αVβ3 and αVβ5, MTT assay was performed using two ovarian cancer cell lines. As shown in Figure 1A, CGT significantly reduced the cell viability of SKOV3 and TOV-21G cells in a dose-dependent manner. Knockdown of integrin αV (ITGAV) and β3 (ITGB3) by shRNA was used to further evaluate the role of integrin αV and β3 in cell growth of SKOV3 cells. The proliferation of SKOV3 shITGAV and shITGB3 cells was significantly lower than SKOV3 shLuc cells (Figure 1B). The rate of proliferation of SKOV3 shITGAVB3 cells, double knockdown of integrin αV and β3, was only slightly lower than SKOV3 shITGAV cells (Figure 1B). Furthermore, the combinational effect of CGT and cisplatin on cell viability of ovarian cancer cells was performed. As shown in Figure 1C, CGT increased cisplatin-induced cytotoxicity in SKOV3 cells. Co-treatment of CGT and cisplatin induced synergistic cytotoxic effects (Figure 1D). To investigate the effect of CGT on cell migration, wound healing assay was performed by Culture-Insert. The migration ability of SKOV3 cells was significantly inhibited by CGT (Figures 1E, F). These results demonstrated that CGT inhibits the cell survival and migration, and enhances cisplatin-mediated cytotoxicity.

**Figure 2:**
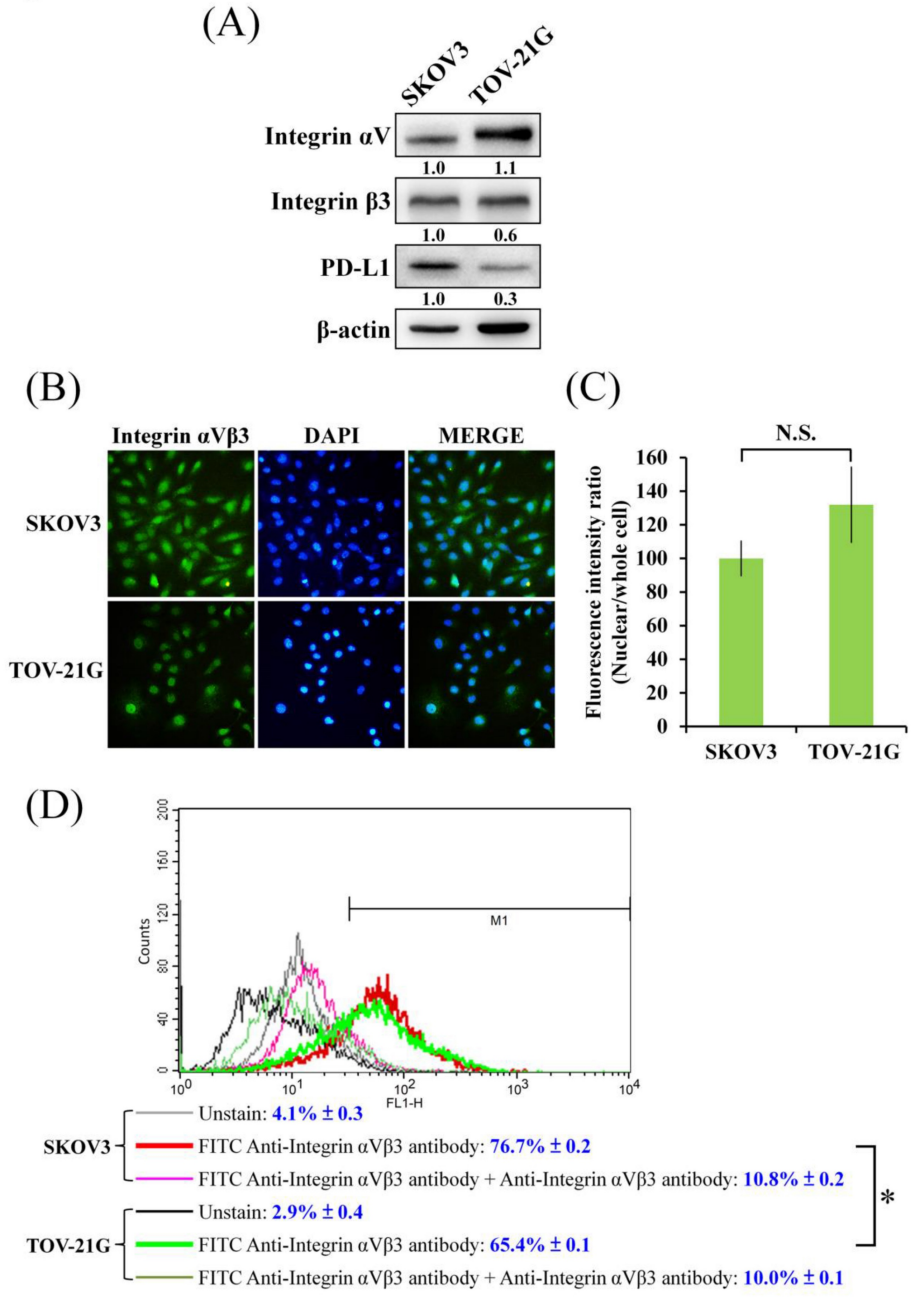
Expression and localization of integrin αVβ3 in ovarian cancer cells. (A) Total cell lysates of SKOV3 and TOV-21G cells (4 × 10^5^ cells of 60 mm dish) were used to investigate the indicated protein expression Western blot assay. (B) SKOV3 and TOV-21G cells were stained with FITC-conjugated integrin αVβ3 antibody and DAPI, and investigate the distribution of integrin αVβ3 in both ovarian cancer cells. (C) Fluorescence intensity of nuclear or whole cell was analyzed by ImageJ. (D) The cells were stained with FITC-conjugated anti-integrin αVβ3 antibody. Non-fluorescent anti-integrin αVβ3 antibody was performed as competing antibody. The unstained and stained cells were analyzed by flow cytometry. The symbol '*' indicates P < 0.05. N.S., not significant.

In Figure 1A, different level of cell viability inhibition by CGT between SKOV3 and TOV-21G cells was found. Because integrin αVβ3 is one of the major targets of CGT; therefore, the protein expressions of integrin αV and β3 were detected to elucidate the difference. As shown in Figure 2A, without standardized by the β-actin, the protein level of integrin αV in SKOV3 cells was lower than that in TOV-21G cells, and there was no marked difference in the expression of integrin β3 between SKOV3 and TOV-21G cells. However, after standardized by the β-actin, the protein level of integrin αV is no marked difference between SKOV3 and TOV-21G cells, but the expression of integrin β3 in TOV-21G cells was lower than it in SKOV3 cells. Immunocytochemistry was performed to further investigate the expression and distribution of integrin αVβ3 in SKOV3 and TOV-21G cells. The integrin αVβ3 heterodimer was differently localized in cells between SKOV3 and TOV-21G cells (Figure 2B). Integrin αVβ3 heterodimer was evenly distributed in SKOV3 cells (Figure 2B). A tendential increase of nuclear-localized integrin αVβ3 was shown in TOV-21G cells compared to SKOV3 cells (Figures 2B, C). Furthermore, the expressions of cell surface integrin αVβ3 in SKOV3 and TOV-21G cells were analyzed by flow cytometry. As shown in Figure 2D, it showed that SKOV3 cells have 10% more expression of surface integrin αVβ3 than TOV-21G cells. These results demonstrated that different expression levels and distribution of integrin αVβ3 heterodimer may make different cytotoxic effects of CGT between SKOV3 and TOV-21G cells.

### 3.2 Integrin αVβ3 inhibition decreases the expressions of downstream protein

**Figure 3:**
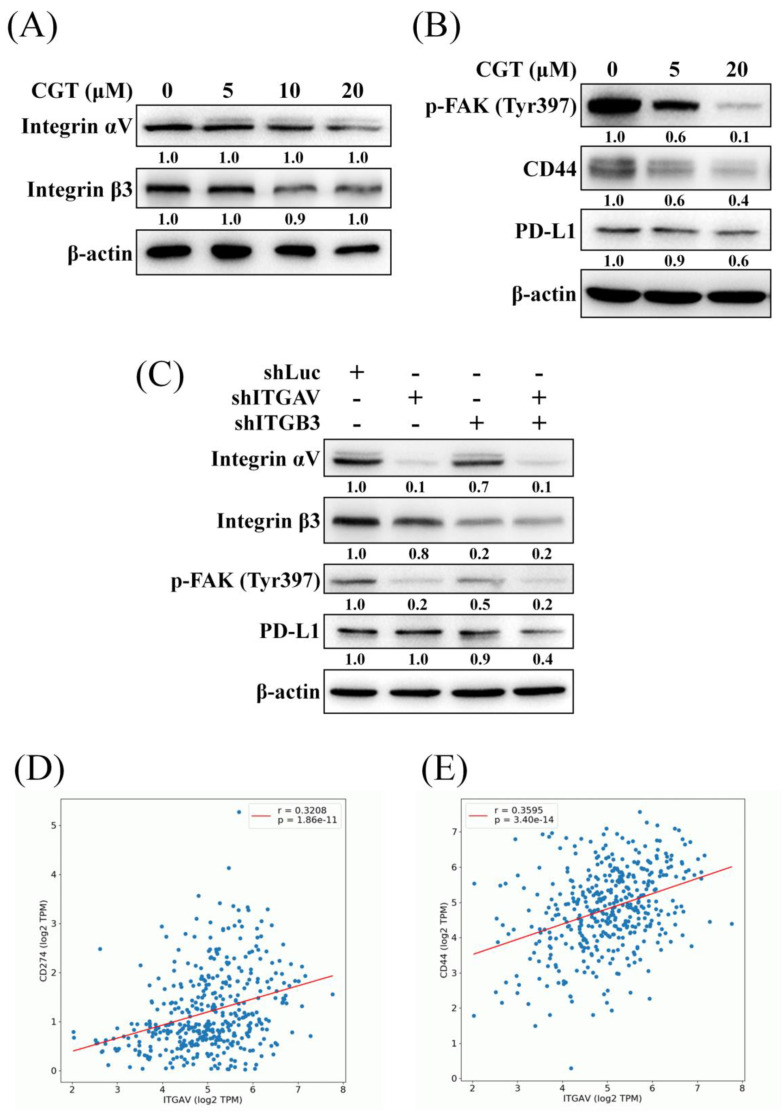
Effect of CGT and knockdown of integrin αVβ3 on expressions of downstream protein. (A) The sample of Figure 3A and Figure 4C are the same. After treatment of various concentrations of CGT (0, 5, 10, and 20 μM) for 48h, total cell lysates of SKOV3 cells (4 × 10^5^ cells of 60 mm dish) were analyzed by Western blot assay to detect the protein expressions of integrin αV and β3. (B) The protein expressions of phospho-FAK, CD44, and PD-L1 in SKOV3 cells after treating with CGT for 48h were analyzed by Western blot assay. (C) Western blot assay was performed to detect the expressions of the indicated protein in SKOV3 shLuc, shITGAV, shITGB3, and shITGAVB3 cells. β-actin served as a loading control. The results showed correlations between the gene expression of integrin αV and (D) CD274 (PD-L1) or (E) CD44 by the OncoDB database.

To investigate the effect of CGT on the expressions of integrin αV and β3 and the downstream protein, the western blot assay was performed. As shown in Figure 3A, CGT did not alter the protein expressions of integrin αV and β3 in SKOV3 cells. CGT inhibited the activity of FAK, the important downstream mediator of integrins, in a dose-dependent manner (Figure 3B). Integrin αVβ3 and CD44 are the receptors of osteopontin (OPN) and crosstalk via regulating signaling pathways 18. The expression of CD44 was reduced by CGT (Figure 3B). In addition, PD-L1, an inhibitory ligand of PD-1 to protect cancer cells from direct attack by cytotoxic T cells, is also regulated by the integrin αVβ3 signaling pathway 19. The result showed that CGT decreases PD-L1 expression in SKOV3 cells (Figure 3B). However, CGT did not reduce but slightly increased the mRNA expressions of CD44 and PD-L1 in SKOV3 cells (Supplementary Figure 1). The above effects on protein expression induced by integrin αVβ3 inhibition were confirmed by knockdown of integrin αV and β3. The efficiency of alone- or double-knockdown of integrin αV and β3 was detected by western blot analysis (Figure 3C). Alone-knockdown of integrin αV or β3 obviously inhibited the phosphorylation of FAK, and double-knockdown did not further decrease the activity of FAK (Figure 3C). Moreover, only double-knockdown of integrin αV and β3 could reduce the expression of PD-L1 (Figure 3C). Positive correlations have been found between the gene expression of integrin αV (ITGAV) and PD-L1 (CD274) or CD44 in ovarian serous cystadenocarcinoma from the OncoDB online database (Figures 3D, E). These results demonstrated that inhibition of integrin αVβ3 decreases the activity of FAK and the expressions of CD44 and PD-L1.

### 3.3 CGT induces apoptosis in SKOV3 cells

**Figure 4:**
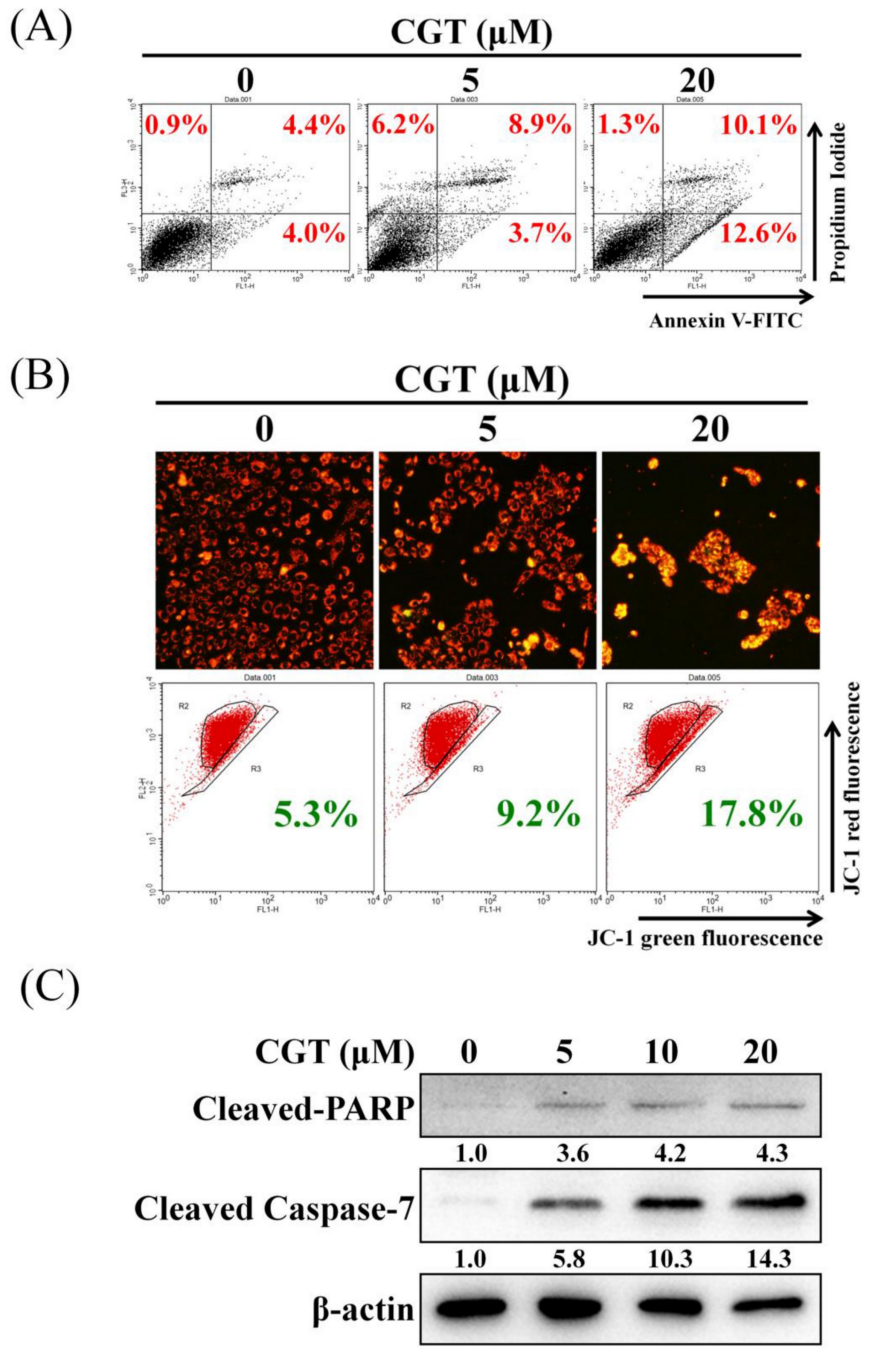
Effect of CGT on apoptosis induction. (A) After treatment with CGT (0, 5, and 20 μM) for 48 h, SKOV3 cells (4 × 10^5^ cells of 60 mm dish) were stained with annexin V-FITC/PI and analyzed by flow cytometry. (B) Mitochondrial membrane potential in SKOV3 cells (4 × 10^5^ cells of 60 mm dish) treated with CGT (0, 5, and 20 μM) for 48 h was analyzed by fluorescence microscope and flow cytometry. (C) The sample of Figure 3A and Figure 4C are the same. The protein expressions of cleaved PARP and cleaved caspase-7 in SKOV3 cells after treating with CGT for 48h were analyzed by Western blot assay. β-actin served as a loading control.

To elucidate the mechanism of cell viability inhibition by CGT, the annexin V/propidium iodide staining assay was performed to investigate cell death. As shown in Figure 4A, CGT induced cell death in SKOV3 cells. The mitochondrial membrane potential was suppressed by CGT in a dose-dependent manner (Figure 4B). The expressions of cleaved PARP and cleaved caspase-7 were increased by CGT (Figure 4C). These results demonstrated that CGT induces apoptotic cell death in SKOV3 cells.

### 3.4 CGT inhibits SKOV3 tumor spheroid formation in extracellular matrix

**Figure 5:**
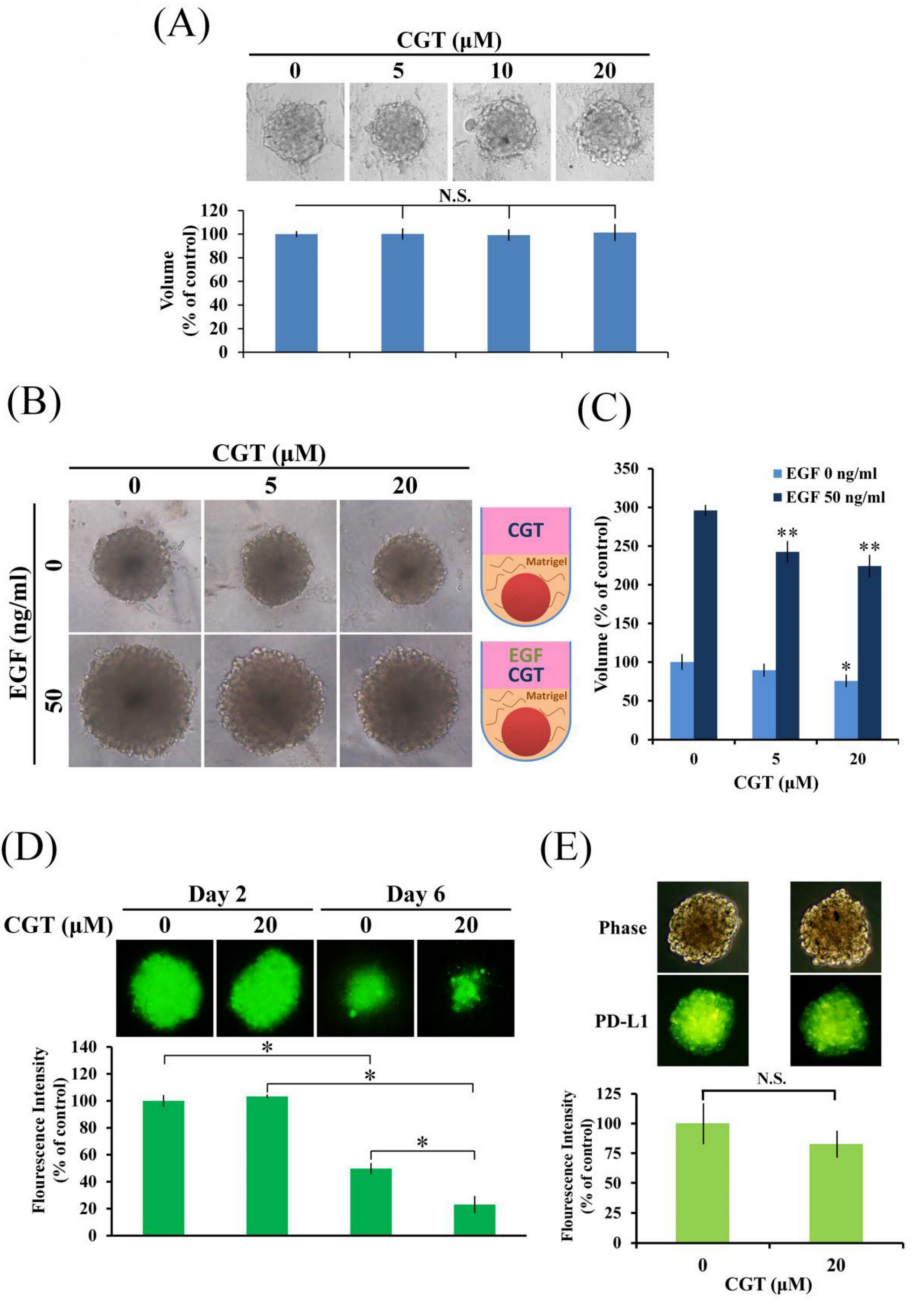
Effect of CGT on tumor spheroid formation. (A) SKOV3 cells (1 × 10^3^ cells/well of 96-well dish) were seeded onto ultra-low attachment 96-well plates. After 96 h incubation for spheroid formation, CGT-containing medium was added to the well, and the spheroids were incubated for another 7 days. The volumes of spheroids were determined by the formula 0.5 × larger diameter × small diameter^2^. Data show the relative spheroid volume, and the volume of spheroid without treatment was set at 100%. (B) Effect of CGT on SKOV3 tumor spheroids growth in 50% Matrigel with or without EGF stimulation. (C) Volume of SKOV3 tumor spheroids in 50% Matrigel treating with CGT and EGF. (D) After 96 h incubation for spheroid formation, the spheroids were subsequently co-cultured with Jurkat T-cells for additional 48 hours in the presence of CGT and 1× T Cell Stimulation Cocktail. (E) Expression of surface PD-L1 in SKOV3 tumor spheroid with or without CGT treatment. The symbol '*' and '**' indicates P < 0.05 and P < 0.001, respectively. N.S., not significant.

CGT has been investigated in *in vitro* models not *in vivo*. To correlate the findings with tumor suppression, 3D tumor spheroid formation assay was performed to investigate drug effects *in vitro*. As shown in Figure 5A, CGT did not alter the size of tumor spheroid of SKOV3 cells. There are also no differences in the size of tumor spheroid formation between SKOV3 shLuc and SKOV3 shITGAVB3 cells (Supplementary Figure 2). Integrins are the receptor of ECM; therefore, we further examine the effect of CGT on tumor spheroid formation in ECM. The volume of SKOV3 tumor spheroids was inhibited by CGT (Figures 5B, C). Furthermore, epidermal growth factor (EGF), an important protein in stimulating tumor growth, was used to promote the growth of SKOV3 tumor spheroids. The size of the spheroids was markedly increased after EGF stimulation, and the EGF-induced spheroid growth was significantly reduced by CGT (Figures 5B, C). In addition, the tumor spheroid model using SKOV3-EGFP cells was performed to investigate the effect of CGT on T cell-mediated cytotoxicity. As shown in Figure 5D, CGT significantly enhanced the inhibiting effect of T cells on SKOV3-EGFP tumor spheroid. Cell surface PD-L1 expression was detected by Alexa Fluor 488 conjucated PD-L1 antibody that specific recognizes extracellular domain of PD-L1. A tendential decrease in spheroid surface PD-L1 expression was shown after CGT treatment (Figure 5E). These results demonstrated that CGT inhibits ECM-mediated tumor spheroid growth and enhances the inhibiting function of T cells on the spheroid.

### 3.5 CGT inhibits cell migration and F-actin formation of SKOV3 tumor spheroid

**Figure 6:**
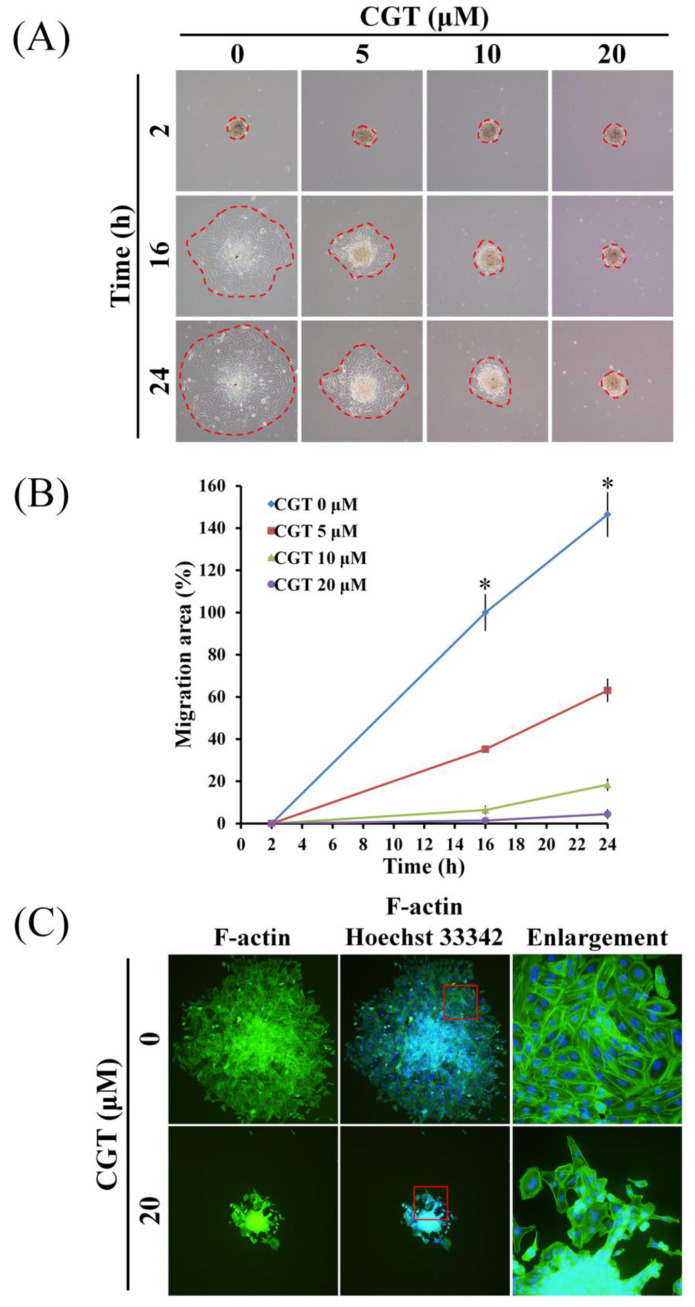
Effect of CGT on cell migration of tumor spheroid. (A) The spheroids with or without CGT treatment were removed to the 6-well plate for attachment and migration. (B) The spreading area of SKOV3 cells migrated from the tumor spheroid was measured by ImageJ software. (C) After attachment for 72h, the tumor spheroid and migrated cells was fixed and stained by phalloidin FITC reagent for detect the F-actin. The symbol '*' indicates P < 0.05.

To investigate the effect of CGT on cell migration of ovarian cancer cells, SKOV3 tumor spheroid-based migration assay was performed. As shown in Figure 6A, CGT markedly reduced the cells migrated from the SKOV3 tumor spheroid. The spreading area of SKOV3 cells migrated from the tumor spheroid was significantly reduced by CGT in a dose-dependent manner (Figure 6B). Formation of F-actin cytoskeleton is important for cell migration; therefore, the F-actin staining assay was performed to investigate the effect of CGT on F-actin formation in SKOV3 cells migrated from the tumor spheroid. CGT decreased the expression of F-actin in the SKOV3 cells migrated from the tumor spheroid. These results demonstrated that CGT decreases the ability of migration of SKOV3 cells using tumor spheroid-based migration assay (Figure 6C).

## 4. Discussion

Integrins play roles in ovarian cancer progression and treatment resistance 20, 21. Integrin αVβ5, but not αVβ3, is activated to protect the ovarian cancer cells from TRAIL-induced cell death 22. MS-275, a HDAC inhibitor, inhibits the tumor spheroid formation of ovarian cancer cells and impacts on Talin‑1‑α5β1‑integrin‑mediated actin cytoskeleton and extracellular matrix protein remodeling 23. CGT inhibits the cancer cell viability mainly via decreasing the cell attachment and inducing apoptosis and anoikis 24-26. The nuclear-localized integrin αVβ3 has been found in high-grade serous ovarian cancer and can partially increase the cell proliferation 27. The nuclear-localized integrin αVβ3 does not affect cell migration, suggesting that nuclear-localized integrin αVβ3 loses its function on cell adhesion 27. The article demonstrated that nuclear-localized integrin αVβ3 performed moonlighting functions in ovarian cancer pathogenesis 27. In this study, CGT decreased the cell viability in both ovarian cancer cell lines; however, the inhibiting effects of CGT on the cell viability of SKOV3 and TOV-21G cells were different (Figure 1A). The difference in inhibiting effect on ovarian cancer cells prompted us to investigate the expression and localization of integrin αV and β3. As shown in Figure 2A, the protein expression of integrin β3 in TOV-21G cells was lower than it was in SKOV3 cells. Furthermore, different from SKOV3 cells, the integrin αVβ3 showed a more nuclear localization and less membrane localization in TOV-21G cells (Figures 2B, C, D). Taken together, we suggested that the expression and localization of integrin αVβ3 may be factors that determine the effectiveness of CGT.

Tumor spheroid is a 3D cell culture model that is a good *in vitro* assay for analyzing drug effects 28-30. A previous study found that CGT does not affect the growth of spheroids of malignant pleural mesothelioma cells 26. Our result was consistent with the results in that article. We found that CGT and double-knockdown of integrin αV and β3 did not alter the size of SKOV3 spheroids (Figure 5A and Supplementary Figure 2). To mimic the ECM effect on integrins during tumor growth, the SKOV3 tumor spheroids were cultured in Matrigel. Interestingly, CGT significantly reduced the size of SKOV3 spheroids cultured in Matrigel with or without EGF stimulation (Figure 5B). Furthermore, we found that CGT slows down the speed of spheroids attachment in a dose-dependent manner during spheroids migration assay (data not shown), suggesting that integrin αVβ3 and αVβ5 expressed on the surface of the SKOV3 spheroids. Taken together, these results suggested that integrins, at least integrin αVβ3 and αVβ5, on the spheroids were not participating in the growth of the floating tumor spheroids. The floating spheroids may be an assay to investigate the compounds specifically targeting integrins.

PD-L1 is a critical protein in cancer cells to inhibit the function of cytotoxic T cells 31. Integrin αVβ3 positively regulates the expression of PD-L1 via activating STAT1 19. CGT enhanced the inhibiting effect of anti-PD-L1 antibody on murine melanoma 32. In the present study, we also found that CGT and double-knockdown of integrin αV and β3 inhibited the expression of PD-L1 in SKOV3 cells (Figures 3B, C). CGT promoted the cytotoxic effect of T cells on SKOV3 tumor spheroids (Figure 5D). The expressions of surface PD-L1 were partially decreased by CGT in the spheroids (Figure 5E). Taken together, we suggested that PD-L1 inhibition by CGT participate in CGT-enhanced T cells-mediated spheroid shrinkage.

## 5. Conclusion

In this study, CGT induced apoptosis and inhibited spheroid migration in ovarian cancer cells. This is the first study that uses 2D and 3D cell culture models to prove that integrin αVβ3 is a potential therapeutic target for ovarian cancer.

## Supplementary Material

Supplementary figures.

## Abbreviations

2D: two-dimensional
3D: three‑dimensional
EGF: epidermal growth factor
ECM: extracellular matrix
