# Supplementary material for: Targeted inhibition of integrin αVβ3 induces cytotoxicity and suppresses migration ability in ovarian cancer cells and tumor spheroids
Source: Int J Med Sci. 2025 Feb 28;22(7):1544–54. doi: 10.7150/ijms.103141 (PMC11905266; doi:10.7150/ijms.103141)
Supplement: Supplementary file 1 — Supplementary figures. [file ijmsv22p1544s1.pdf]

(A) (B)

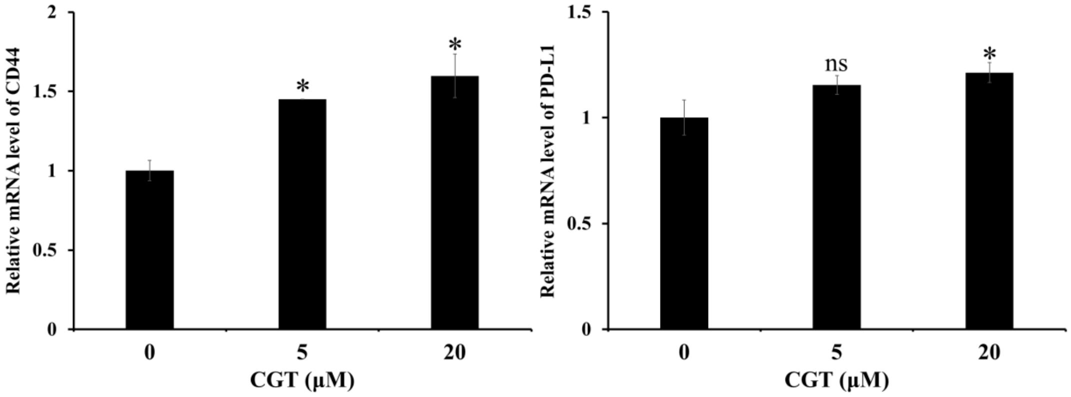

Supplementary Figure 1. Effect of CGT on mRNA expressions of CD44 and PD-L1 in SKOV3 cells

After treatment of CGT for 24 h, the total RNA was collected from SKOV3 cells (4×10^5 cells of 60 mm dish). RT-qPCR was performed to analyze the mRNA expressions of (A) CD44 and (B) PD-L1. The symbol '*' indicates $P < 0.05$; ns, not significant.

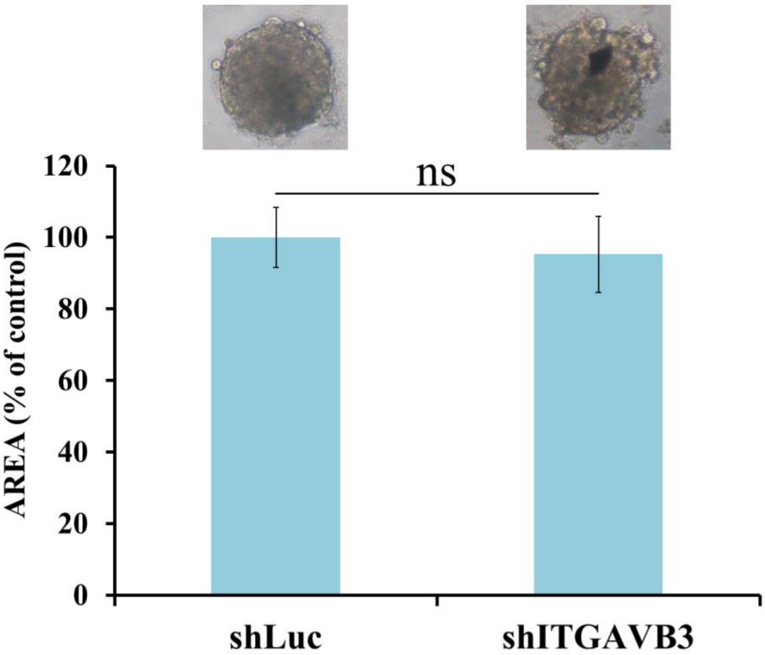

Supplementary Figure 2. Effect of CGT on tumor spheroid formation of SKOV3 shLuc and shITGAVB3 cells

SKOV3 shLuc and shITGAVB3 cells (1×10^3 cells/well of 96-well dish) were seeded onto ultra-low attachment 96-well plates. After 11 days incubation for spheroid formation, the areas of spheroids were determined. Data show the relative spheroid volume, and the area of spheroid of SKOV3 shLuc cells was set at 100%. The symbol ‘ns’ indicates not significant.
